# Decreased Endomorphin-2 and μ-Opioid Receptor in the Spinal Cord Are Associated with Painful Diabetic Neuropathy

**DOI:** 10.3389/fnmol.2016.00080

**Published:** 2016-09-07

**Authors:** Zhen-Zhen Kou, Fa-Ping Wan, Yang Bai, Chun-Yu Li, Jia-Chen Hu, Guo-Tao Zhang, Ting Zhang, Tao Chen, Ya-Yun Wang, Hui Li, Yun-Qing Li

**Affiliations:** ^1^Department of Anatomy and K.K. Leung Brain Research Centre, The Fourth Military Medical UniversityXi’an, China; ^2^Collaborative Innovation Center for Brain Science, Fudan UniversityShanghai, China

**Keywords:** painful diabetic neuropathy, endomorphin 2, μ-opioid receptor, spinal cord, rat

## Abstract

Painful diabetic neuropathy (PDN) is one of the most common complications in the early stage of diabetes mellitus (DM). Endomorphin-2 (EM2) selectively activates the μ-opioid receptor (MOR) and subsequently induces antinociceptive effects in the spinal dorsal horn. However, the effects of EM2-MOR in PDN have not yet been clarified in the spinal dorsal horn. Therefore, we aimed to explore the role of EM2-MOR in the pathogenesis of PDN. The main findings were the following: (1) streptozotocin (STZ)-induced diabetic rats exhibited hyperglycemia, body weight loss and mechanical allodynia; (2) in the spinal dorsal horn, the expression levels of EM2 and MOR decreased in diabetic rats; (3) EM2 protein concentrations decreased in the brain, lumbar spinal cord and cerebrospinal fluid (CSF) in diabetic rats but were unchanged in the plasma; (4) the frequency but not the amplitude of spontaneous excitatory postsynaptic currents (sEPSCs) was significantly higher in diabetic rats than in control rats; and (5) intrathecal injection of EM2 for 14 days in the early stage of PDN partially alleviated mechanical allodynia and reduced MOR expression in diabetic rats. Our results demonstrate that the EM2-MOR signal may be involved in the early stage of PDN.

## Introduction

Diabetic neuropathy is the most common complication of diabetes mellitus (DM). It is known that painful diabetic neuropathy (PDN) in diabetic patients and animal models occurs at the early stage of the disease prior to the appearance of overt clinical symptoms (Ziegler, 2008). In previous studies, streptozotocin (STZ)-induced type 1 diabetic rats developed PDN as early as 2 weeks after STZ injection (Malcangio and Tomlinson, 1998; Kou et al., 2014; Castelli et al., 2016). However, the mechanism underlying PDN remains unclear, and effective drugs for PDN management in diabetes remain unavailable (Jaspan et al., 1983; Bouhassira et al., 2014; Schreiber et al., 2015). Although PDN could benefit from opioid-based therapies, the clinical use of opioids has been hampered by significant side effects, such as gastrointestinal complications, respiratory depression, tolerance and dependence with long-term use (Zhao et al., 2010; Wu et al., 2011; Banafshe et al., 2014). Therefore, searching for more selective and effective therapeutics with fewer side effects is important for treating PDN.

As endogenous opioid peptides, the endomorphins (EMs), EM1 (Tyr-Pro-Trp-Phe-NH2) and EM2 (Tyr-Pro-Phe-Phe-NH2) are highly selective for the μ-opioid receptor (MOR; Champion et al., 1997; Hosohata et al., 1998; Wang et al., 2003). Both EM1 and EM2 have been suggested to be naturally occurring peptides with remarkable antinociceptive properties and less adverse effects than opioids (Czapla et al., 2000; Varamini and Toth, 2013). Importantly, previous studies have confirmed that morphine and EM differentially regulated MOR mRNA expression and function (McConalogue et al., 1999; Yu et al., 2003), suggesting that EMs might provide excellent therapeutic potential benefits as replacements for morphine-like opioids.

It has been shown that EM1-immunoreactivity (-ir) is primarily restricted to the brain, but EM2-ir is found primarily in the superficial layer of the spinal dorsal horn, which is considered to be the fundamental area in nociceptive transmission modulation in the central nervous system (CNS; Martin-Schild et al., 1998, 1999; Pierce and Wessendorf, 2000). Importantly, a significant effect of regulating nociceptive transmission of EM2 has been documented in the spinal cord (Labuz et al., 2003). In the spinal dorsal horn, EM2 plays a more efficient analgesic role than that of EM1 in several animal models of pain, including neuropathic pain (Tseng et al., 2000). In response to EM2, the antinociceptive effect is blocked by MOR antagonists, and EM2 treatment fails to induce significant antinociceptive effects in MOR knockout mice, indicating that the antinociceptive activities of EM2 are restricted to the MOR in the spinal cord (Narita et al., 1999; Tseng et al., 2000). Therefore, we hypothesized that EM2 and MOR may be involved in PDN in the spinal cord.

It has been noted that the level of peripheral EMs was suppressed in diabetic patients compared with healthy volunteers (Xia et al., 2010). In the type 1 diabetic mouse, previous results have demonstrated that the inhibitory effects of EM2 on colonic motility were attenuated, but the effects of EM1 were unchanged, indicating that EM2 may play a more important role in diabetes (Wang et al., 2008). Reports have suggested that the effects of EM2 were reduced in PDN (Chen et al., 2002), and this result may be due to decreased EM2, MOR or both. However, the underlying mechanism of EM2 and MOR in PDN is still unclear.

Based on these considerations, we address the hypothesis that EM2 and MOR may be associated with the pathogenesis of PDN in the spinal dorsal horn. Changes in EM2 and MOR were examined in diabetic rats by immunohistochemistry, enzyme-linked immunosorbent assay (ELISA), Western blotting and electrophysiological techniques. In addition, we applied intrathecal infusions of EM2 for 14 days in diabetic rats. We attempted to test the hypothesis that the EM2-MOR signal may be associated with the development of PDN.

## Materials and Methods

### Experimental Animals

All animal studies were conducted using approved protocols and carried out in accordance with the Principles of Laboratory Animal Care (NIH Publication no. 85-23, revised 1985). The Animal Care and Use Committees of the Fourth Military Medical University reviewed and approved all protocols. Male *Sprague-Dawley* rats weighing 220–250 g purchased from Laboratory Animal Resources of the Fourth Military Medical University were utilized and given a single intraperitoneal injection of STZ (60 mg/kg, Sigma, St. Louis, MO, USA), which was freshly dissolved in ice-cold sodium citrate (pH 4.5). Diabetes was confirmed on the 3rd day by measurements of blood glucose concentrations in samples obtained from the tail vein using a strip-operated reflectance meter (Active; Roche Diagnostics, Mannheim, Germany). Only rats with blood glucose concentrations greater than 20 mM were further used. Citrate buffer-treated rats were used as a normoglycemic control (blood glucose less than 12 mM). All animals were housed in standard conditions (12 h light/dark cycles) with water and food available *ad libitum*.

### Measurement of Hindpaw Withdrawal Threshold

Experiments were performed on the STZ-treated rats and saline-treated control rats, according to our protocols reported previously (Mei et al., 2011; Kou et al., 2013, 2014). To quantify the mechanical sensitivity, rats were placed in individual plastic boxes and allowed to acclimate for 30 min. A series of calibrated von Frey filaments (Stoelting, Kiel, WI, USA) ranging from 0.4 to 60.0 g were applied to the plantar surface of the hindpaw of rats with a sufficient force to bend the filaments for 5 s or until the paw withdrew. There was a 15 s interval between applications that allowed the animal to return to a relatively inactive position. In the presence of a response, the filament of next lower force was applied. The filament of next greater force was applied when a response was absent. A positive response was determined by a sharp withdrawal of the paw. Each filament was applied 10 times, and the minimal value that caused a response at least six times was recorded as the paw withdrawal threshold (PWT). All behavioral studies were performed under blind conditions.

### Immunohistochemistry

Rats (*n* = 6 in each group) were deeply anesthetized by an intraperitoneal injection of pentobarbital (50 mg/kg, i.p.) and perfused for immunohistochemistry. After perfusion, lumbar segments (L4–L6) of the spinal cord were removed, post-fixed and placed in 30% (w/v) sucrose solution for 24 h at 4°C. Transverse sections of the spinal cord (25 μm) were incubated in blocking solution (5% v/v normal goat serum) for 1 h at room temperature and then incubated overnight at 4°C with primary antibodies, rabbit anti-EM2 (1:200; Chemicon, Temecula, CA, USA) and guinea pig anti-MOR (1:500; Abcam, Cambridge, MA, USA). Then, the sections were washed with phosphate-buffered saline (PBS) and incubated with Alexa488-conjugated donkey anti-rabbit IgG (1:500; Invitrogen, Carlsbad, CA, USA) and Alexa594-conjugated donkey anti-guinea pig IgG (1:500; Invitrogen) for 6 h. Finally, the sections were rinsed with PBS, mounted onto clean glass slides, air-dried and coverslipped with a mixture of 0.05 M PBS containing 50% (v/v) glycerin and 2.5% (w/v) triethylenediamine. The sections were viewed under a confocal laser scanning microscope (FV-1000, Olympus, Tokyo, Japan). The images were captured and analyzed using Fluoview 1000 (Olympus).

### ELISA

Experiments were carried out according to a previous protocol (Marténez-Lorenzana et al., 2008). Brain, lumbar spinal cord, cerebrospinal fluid (CSF) and plasma samples were collected to quantify the EM2 concentration with different experimental conditions. CSF (*n* = 6 in each group) was taken from the cisterna magna at different times, and the samples were stored at −70°C for subsequent analyses. After CSF samples were obtained, 1.0 ml of blood was collected from the heart and stored in chilled Eppendorf tubes containing 1.0 mg EDTA. Blood samples were centrifuged at 0°C, 1600 rpm for 15 min, and the plasma was harvested and stored at −70°C. Then, tissues from the lumbar spinal cord (L4–L6) and brain from each rat were removed and homogenized in a solution of 50 mM Tris–HCl (pH 9) containing protease inhibitors. After centrifugation for 30 min at 4°C, the supernatants were collected, and the protein content was determined. EM2 concentrations in the brain, lumbar spinal cord, CSF and plasma samples were analyzed using an enzyme immunoassay (EIA) kit (Phoenix Pharmaceuticals, Inc., EK-044-11). For each EIA sample for each rat, 100 μl of CSF was used. The instructions supplied with the EM2 EIA kit were followed without modification, and EM2 was measured directly in the plasma, CSF, brain and spinal cord tissue samples. The data from a microplate ELISA reader were analyzed using an ELISA reader (Bio-Rad, iMark, Hercules, CA, USA).

### Western Blotting

Rats (*n* = 6 in each group) were killed under pentobarbital anesthesia. The lumbar spinal dorsal horn and the L4–L6 dorsal root ganglions (DRGs) were homogenized in lysis buffer containing proteinase inhibitors and phosphatase inhibitors (Roche, Switzerland). Then, the homogenized samples were centrifuged at 12,000× g for 10 min at 4°C. Next, the lysate protein concentrations were determined with a BCA protein assay kit (Pierce, Appleton, WI, USA), mixed with 5× sodium dodecyl sulfate (SDS) sample buffer, and boiled for 10 min. Equal samples of protein from animals were electrophoresed by SDS-PAGE in 12% polyacrylamide gel and transferred to PVDF membranes (Millipore, Bedford, MA, USA). Then, membranes were blocked with 5% bovine serum albumin (BSA) and incubated overnight at 4°C with primary antibodies, including anti-MOR (rabbit polyclonal, 1:500; Abcam), anti-phosphorylated-MOR (Ser375, rabbit polyclonal, 1:1000; Cell Signaling Technology, Beverly, MA, USA) and anti-β-actin (mouse monoclonal, 1:2000; Sigma). After incubation of the membrane with peroxidase-conjugated anti-rabbit secondary antibodies (Santa Cruz Biotechnology, Santa Cruz, CA, USA) for 2 h at room temperature, the reaction products were visualized with enhanced chemiluminescence (Amersham Life Science, Amersham, UK). Western blots were made in triplicate. Band density was measured and normalized against a loading control band.

### Intrathecal Injection

Intrathecal implantation was performed as our reports have previously described (Mei et al., 2011; Kou et al., 2013). The polyethylene (PE) tube (Becton Dickinson and Company, Franklin Lakes, NJ, USA) was inserted directly into the subarachnoid space of the lumbar enlargement (L4–L6). Briefly, under pentobarbital anesthesia, a midline incision was made at the back of the rat. A pre-measured length of PE10 tube (I.D. 0.28 mm and O.D. 0.61 mm) was passed caudally at the level of the lumbar vertebra. After administration of 2% lidocaine (10 μl) through the intrathecal catheter, only rats that showed complete paralysis of the tail and bilateral hind legs and full recovery from the paralysis 30 min after lidocaine injection were judged to be neurologically normal. After tube implantation, rats were allowed to recover for 2 days. Rats were intrathecally injected with either EM2 (20 μg) or 10 μl saline for a control through the catheter from 10:00 am to 10:30 am per day for 14 days (Zadina et al., 1997; Horvath et al., 1999).

### Electrophysiological Study

#### Slice Preparation

Fourteen days after STZ injection, rats with significant mechanical allodynia were anesthetized with 7% chloralic hydras and then perfused transcardially for 2 min with 100 ml of ice-cold sucrose-substituted artificial CSF (sucrose ACSF: 220 mM sucrose, 2.5 mM KCl, 0.5 mM CaCl_2_, 6.0 mM MgSO_4_·7H_2_O, 1.2 mM NaH_2_PO_4_, 26 mM NaHCO_3_, 10 mM glucose, 1 mM ascorbate and 3.0 mM pyruvate, 290–330 mOsm, pH 7.25–7.45; Chen et al., 2015a). A laminectomy was performed to remove the lumbar spinal cord. Transverse slices of L4–L5 spinal segments (400 μm) were cut on a vibrating microtome (Leica VT 1200s, Heidelberger, Nussloch, Germany) in ice-cold sucrose ACSF. Then, the slices were collected in an incubation chamber filled with normal ACSF (124 mM NaCl, 2.5 mM KCl, 2 mM MgSO_4_·7H_2_O, 2 mM CaCl_2_, 1 mM NaH_2_PO_4_, 25 mM NaHCO_3_, 25 mM glucose, 1 mM ascorbate and 3.0 mM pyruvate) and incubated for 1 h at room temperature (22–25°C). All the ACSF solutions in this experiment were continuously equilibrated with the carbogen gas (95% O_2_ and 5% CO_2_).

#### Electrophysiological Recordings

Each spinal cord slice was transferred to recording chambers, fixed with parallel nylon threads, and supported by a U-shaped platinum weight. The slice was continuously perfused with ACSF at 2–3 ml/min. Neurons in the lamina II of the spinal cord slice were identified with differential interference contrast/infrared illumination on a fixed-stage microscope (BX51W1; Olympus, Tokyo, Japan). Voltage-clamp recording procedures were used as described previously (Yang et al., 2001; Chen et al., 2011, 2015a). Currents were recorded from lamina II neurons at a holding potential of −70 mV using an internal pipette solution containing the following reagents: 130 mM potassium gluconate, 5 mM NaCl, 15 mM KCl, 0.4 mM EGTA, 10 mM HEPES, 4 mM Mg-ATP and 0.2 mM Tris-GTP. The solution was adjusted to pH 7.2–7.4 with 1 M KOH, and the osmolality was adjusted to 290–300 mOsm. Recordings of spontaneous excitatory postsynaptic currents (sEPSCs) began approximately 10–15 min after whole-cell access was established and the current reached a steady state. The input resistance was monitored, and the recording was abandoned if it changed by more than 15%. All signals were recorded using a MultiClamp 700B amplifier (Axon Instruments, Forster City, CA, USA). The data were recorded using the software pClAMP 10.2 (Axon Instruments). To examine the effects of EM2 on sEPSCs, a concentration of 3 μM EM2 (Sigma) was perfused through the bath solution (Chen et al., 2015a).

### Statistical Analyses

The results from immunohistochemistry were obtained as detailed in previous reports (Fernyhough et al., 1989; Aizawa and Eggermont, 2006; Zuo et al., 2011; Kou et al., 2013). In the spinal dorsal horn, the relative optical density (ROD, the control group was set as 100%) was used for statistical analysis as in our previous reports (Zuo et al., 2011; Kou et al., 2013, 2014). Analysis of the time course of behavior tests between saline- and EM2-treated groups was performed by a two-factor (group and time) repeated-measures analysis of variance (ANOVA). The data were expressed as the mean ± SD and were analyzed by SPSS 16.0 (SPSS Inc., Chicago, IL, USA). The data from Western blotting and ELISA were analyzed with a one-way ANOVA with the Student–Newman–Kuels (SNK) *post hoc* test. The results from patch-clamp recordings were presented as the mean ± SEM and analyzed as previously reported (Kohno et al., 2005; Chen et al., 2011, 2015a). The frequency and amplitude of sEPSCs were analyzed using a peak detection program (MiniAnalysis; Synaptosoft, Decatur, GA, USA). The cumulative probability of the amplitude, time decay and inter-event interval of sEPSCs was compared by using the Komogorov-Smirnov test. The effects of EM2 on sEPSCs were determined using a paired or unpaired Student’s *t*-test, and *p* < 0.05 was considered statistically significant.

## Results

### STZ-Induced Type 1 Diabetic Rats

**Figure 1:**
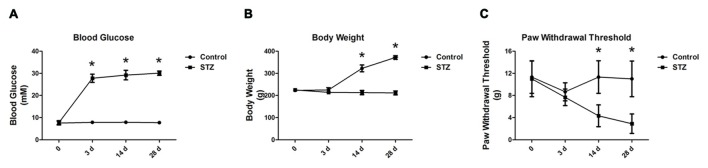
**Streptozotocin (STZ)-induced diabetic rats exhibit elevated blood glucose concentration, reduced body weight and decreased paw withdrawal thresholds (PWTs).** Changes in blood glucose concentration **(A)**, body weight **(B)** and PWTs **(C)** in rats after STZ injection from day 3 to day 28. Mean ± SD, *n* = 6 per group, *p < 0.05 vs. non-diabetic control rats.

Following an injection of STZ, we examined changes in the blood glucose and body weight of rats for 28 days. There was no difference in basal blood glucose concentration between STZ-treated rats and control rats at the onset of the study (7.55 ± 0.83 mM vs. 7.75 ± 0.86 mM in the control group, *p* > 0.05, Figure 1A). On the 3rd day, compared with control rats, rats treated with STZ exhibited significant hyperglycemia (27.78 ± 1.88 mM vs. 7.88 ± 0.69 mM in the control group, *p* < 0.05) until day 28 (30.07 ± 1.00 mM vs. 7.75 ± 0.49 mM in the control group, *p* < 0.05, Figure 1A). Additionally, the body weights of the diabetic rats were significantly lower than those of the control rats on day 14 (213.33 ± 9.83 g vs. 322.50 ± 15.41 g in the control group, *p* < 0.05) and continued thereafter until day 28 (211.67 ± 8.76 g vs. 371.67 ± 8.16 g in the control group, *p* < 0.05, Figure 1B). The results indicate that rats injected with STZ developed type 1 diabetic features including hyperglycemia and body weight loss.

### Mechanical Allodynia in STZ-Induced Type 1 Diabetic Rats

Fourteen days after diabetes onset, when tested with von Frey filaments, the PWTs in diabetic rats were significantly lower than those in control rats (4.33 ± 1.97 g vs. 11.33 ± 2.94 g in the control group, *p* < 0.05, Figure 1C). On day 28, there was a significant difference between the diabetic rats and controls (2.90 ± 1.76 g vs. 11.00 ± 3.22 g in the control group, *p* < 0.05, Figure 1C). The behavioral results suggest that mechanical allodynia developed in STZ rats on day 14 but peaked on day 28, along with an increased blood glucose level and reduced body weight (Figure 1C).

### Changes in EM2 and MOR in the Spinal Dorsal Horn of Diabetic Rats

**Figure 2:**
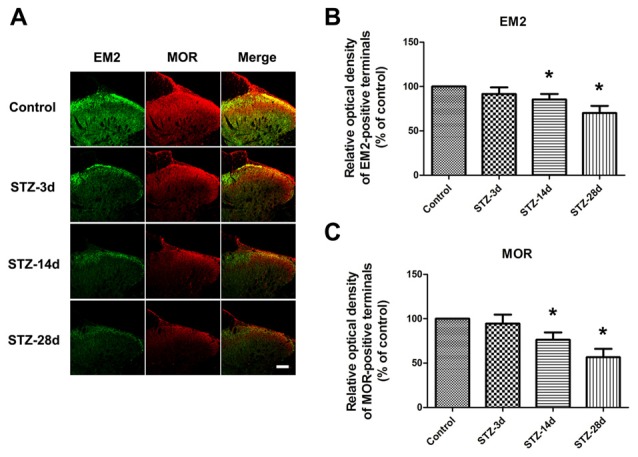
**Photomicrographs indicate the localization of endomorphin-2 (EM2) and μ-opioid receptor (MOR) in the spinal cord.** Fluorescent photographs show how EM2-immunopositive (IP) and MOR-IP terminals are distributed in the spinal dorsal horn **(A)**. Scale bar: 100 μm in **(A)**. Statistical analysis of the relative optical density (ROD) of EM2-IP terminals **(B)** and MOR-IP terminals **(C)** in different groups. The sample of the control group was set at 100%. Mean ± SD, *n* = 6 per group, *p < 0.05 vs. non-diabetic control rats.

First, we investigated the expression of EM2 and MOR in the spinal cord during the progressive mechanical allodynia by immunohistochemistry. The EM2-positive terminals were densely observed in the superficial layer of the spinal dorsal horn (Figure 2A). Our results showed that EM2-positive puncta in laminae I and II of the spinal cord in diabetic rats decreased gradually but significantly from day 14 (85.4% of control, *p* < 0.05) to day 28 (70.1% of control, *p* < 0.05, Figures 2A,B). In the spinal cord, MOR-labeling was also found in the dorsal horn (Figure 2). Accompanied by the reduced expression of EM2, there was a marked decrease in MOR-positive terminals on day 14 (76.3% control, *p* < 0.05) and on day 28 (56.6% of control, *p* < 0.05, Figures 2A,C) in the spinal dorsal horn.

**Figure 3:**
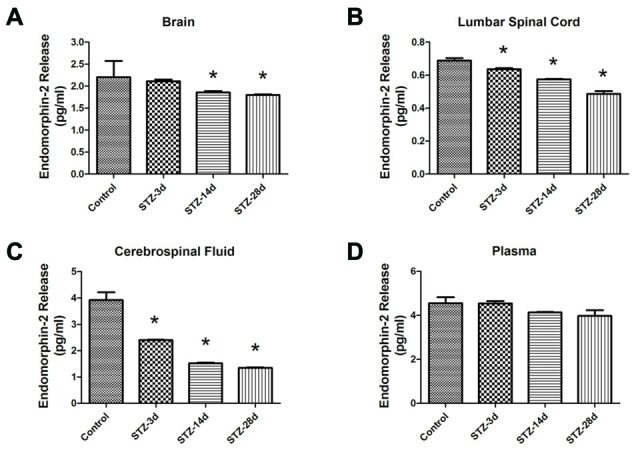
**EM2 concentrations in the brain (A), lumbar spinal cord (B), cerebrospinal fluid (CSF; C) and plasma (D) in control and diabetic rats.** Values are expressed as pg/ml. Mean ± S.E.M., *n* = 6 per group, *p < 0.05 vs. non-diabetic control rats.

Second, we observed the changes in EM2 concentration in the brain, lumbar spinal cord, CSF and plasma in diabetic rats by ELISA. The results showed that diabetic rats had markedly lower EM2 concentrations in brain tissue on day 14 (1.86 ± 0.03 pg/ml) and day 28 (1.80 ± 0.02 pg/ml) compared with the control group (2.21 ± 0.37 pg/ml, *p* < 0.05, Figure 3A). In the lumbar spinal cord, the concentrations of EM2 significantly decreased in diabetic rats: 0.64 ± 0.01 pg/ml on day 3 (*p* < 0.05), 0.58 ± 0.002 pg/ml on day 14 (*p* < 0.05) and 0.49 ± 0.02 pg/ml on day 28 (*p* < 0.05, Figure 3B). As demonstrated in Figure 3C, EM2 concentrations in CSF decreased substantially in diabetic rats on day 3 compared with controls (2.40 ± 0.02 pg/ml, *p* < 0.05) and remained at the reduced level on day 28 (1.52 ± 0.03 pg/ml, *p* < 0.05). No significant variation in EM2 concentration was observed in the plasma (*p* > 0.05, Figure 3D).

**Figure 4:**
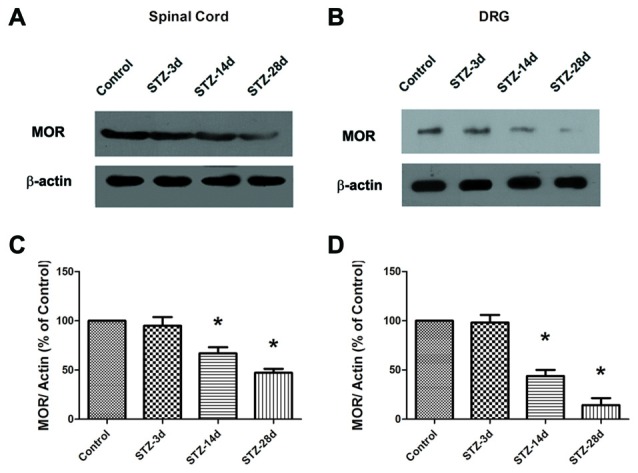
**The expression of MOR was detected in the spinal cord (A) and the dorsal root ganglion (DRG; B) by Western blotting.** Statistical analysis of MOR in the spinal cord **(C)** and the DRG **(D)** in different groups. The sample of the control group was set at 100%. Mean ± SD, *n* = 6 per group. *p < 0.05 vs. non-diabetic control rats.

Third, the data from Western blotting indicated a significant decrease in MOR expression from day 14 in the spinal cord of diabetic rats (67.1% of control, *p* < 0.05, Figures 4A,C). Compared with the controls, the diabetic rats showed a reduced level of MOR on day 28 (47.3% of control, *p* < 0.05, Figures 4A,C). Because MORs in the spinal dorsal horn mainly originate from the central axons of the DRG, we also examined the changes in MOR in the DRG. The expression of MOR decreased significantly from day 14 (43.9% of control, *p* < 0.05, Figures 4B,D) to day 28 (14.3% of control, *p* < 0.05, Figures 4B,D). These results suggest that the decrease in EM2 and MOR in the spinal cord was consistent with the progressive mechanical allodynia in diabetic rats.

### Effects of Intrathecal Injection of EM2 on Mechanical Allodynia

**Figure 5:**
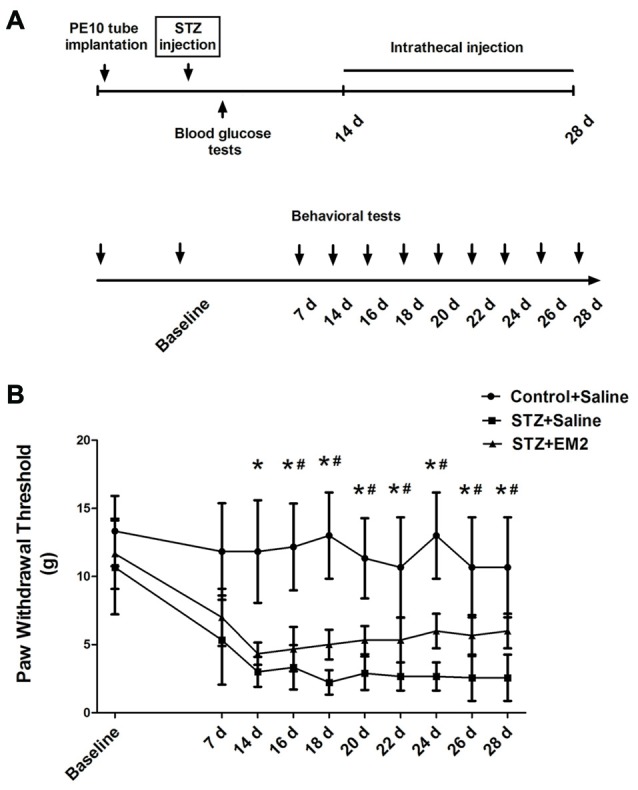
**Effects of intrathecal EM2 on mechanical sensitivity in diabetic rats compared with the controls.** PWTs were measured before (baseline), day 7, 14, 16, 18, 20, 22, 24, 26 and 28 **(A)**. Intrathecal injection of EM2 from day 14 to day 28 alleviated the mechanical allodynia **(B)** in the diabetic rat. Mean ± SD, *n* = 6 per group, *p < 0.05 vs. STZ rats-saline group at corresponding time points, ^#^*p* < 0.05 vs. control-saline group at corresponding time points.

To detect the effects of EM2 on PDN, EM2 was injected intrathecally once a day from day 14 to day 28 in the STZ-induced type 1 diabetic rats. According to previous reports (Zadina et al., 1997; Janecka et al., 2008), EM2 (20 μg) or saline was applied through intrathecal injection for 14 days. The PWTs were examined within 30 min after injection, from day 7 to day 28 (Figure 5A). After EM2 treatment, the PWTs in diabetic rats were elevated significantly on day 16 (*p* < 0.05, compared with that of the STZ-saline group, Figure 5B). Moreover, 14-day treatment with EM2 apparently increased PWTs in diabetic rats on day 28 (6.00 ± 1.26 g vs. 2.57 ± 1.70 g in STZ-saline group, *p* < 0.05, Figure 5B). However, we found that intrathecal administration of EM2 could not fully attenuate mechanical allodynia in diabetic rats compared with control rats treated with saline (*p* < 0.05, Figure 5B).

**Figure 6:**
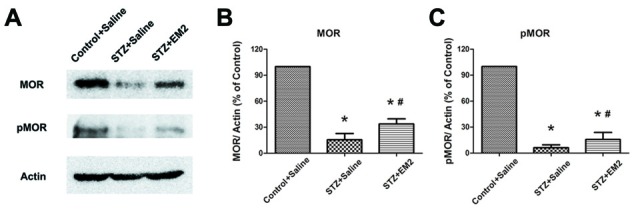
**Effects of intrathecal EM2 on MOR and pMOR in the spinal dorsal horn.** Intrathecal injection of EM2 attenuated the reduction in MOR and pMOR after 14 days of administration **(A)**. Statistical analysis of MOR **(B)** and pMOR **(C)** in different groups. The sample of the control group was set at 100%. Mean ± SD, *n* = 6 per group, *p < 0.05 vs. STZ rats-saline group at corresponding time points, ^#^*p* < 0.05 vs. control-saline group at corresponding time points.

After EM2 administration, the results from Western blotting demonstrated that intrathecal EM2 treatment significantly increased the expression of MOR in the spinal cord of diabetic rats (*p* < 0.05, compared with that of the STZ-saline group, Figures 6A,B). Moreover, the tendency for reduced pMOR levels was partially attenuated after EM2 treatment (*p* < 0.05, compared with that of the STZ-saline group, Figures 6A,C), suggesting that EM2 application attenuated the down-regulation of MOR in the spinal cord and alleviated mechanical allodynia in STZ-induced type 1 diabetic rats.

### Effects of EM2 on Glutamatergic Input to Spinal Dorsal Horn Neurons in Diabetic Rats

**Figure 7:**
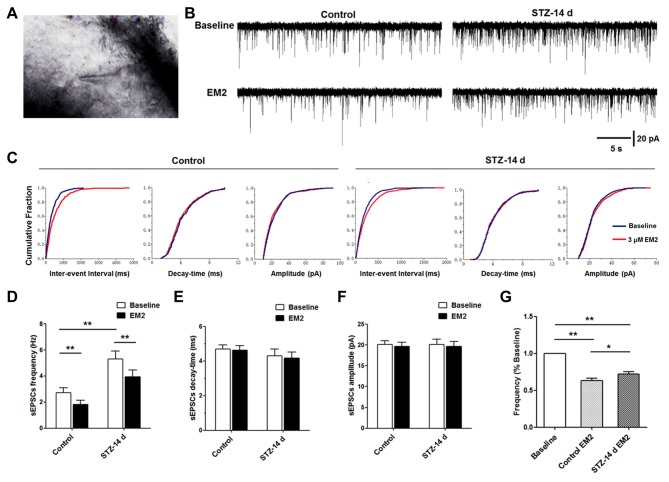
**Effects of EM2 on glutamatergic spontaneous excitatory postsynaptic currents (sEPSCs) of neurons in spinal lamina II in the control and diabetic rats.** A neuron of lamina II pictured during whole-cell patching **(A)**. The illustrated representative traces of sEPSCs at baseline, with application of 3 μM EM2 in a neuron of lamina II from a control and a diabetic rat **(B)**. Cumulative fraction of the inter-event interval, time decay and amplitude of sEPSCs **(C)** during baseline (blue) and application of EM2 (red) in the control and diabetic rats in the same neurons shown in **(B)**. Analyses of all data in 15 neurons from six control rats and 14 neurons from 10 diabetic rats illustrated that the frequency was significantly higher in the diabetic rats **(D)**, but there were no differences in decay time **(E)** and amplitude **(F)** between the two groups of rats. Application of EM2 decreased the frequency **(D)** but not the time decay **(E)** or the amplitude **(F)** of the sEPSCs. The bar chart shows that the frequency of sEPSCs was significantly reduced by EM2, but the inhibitory effects of EM2 were lower in diabetic rats **(G)**. Mean ± SEM, *p < 0.05, **p < 0.01.

MOR localized in primary central terminals is essential for EM2 analgesia in the spinal cord (Sakurada et al., 1999, 2001; Chen et al., 2015b). One of the mechanisms is presynaptic inhibition of the release of neurotransmitters, especially the release of glutamate (Fujita and Kumamoto, 2006; Fichna et al., 2007). In the present study, we found that the expression of MOR was down-regulated in the DRG neurons and spinal cord. Next, to determine the consequence of the reduced MOR exerted on glutamate release, we compared the effects of EM2 on recordings of sEPSCs in lamina II neurons (Figure 7A). The results indicated that the baseline frequency of glutamatergic sEPSCs was significantly higher in diabetic rats than in control rats (*p* < 0.05, 5.31 ± 0.6 Hz vs. 2.72 ± 0.39 Hz, unpaired Student’s *t*-test, Figures 7B,D). Perfusion of 3 μM EM2 resulted in a rightward shift in the distribution of the inter-event interval of glutamatergic sEPSCs in the two groups (control rats: 1.82 ± 0.32 Hz; diabetic rats: 3.94 ± 0.52 Hz, Figures 7C,D). However, the amplitude and the decay time between the sEPSCs of control and diabetic rats did not change significantly (*p* > 0.05, paired Student’s *t*-test, Figures 7C,E,F). These results suggest that EM2 decreased the frequency, but not the amplitude of sEPSCs in lamina II neurons. Because the baseline frequency of sEPSCs in control rats was significantly different from the diabetic rats (Figure 7D), we normalized the effect of EM2 to the baseline of sEPSCs (post-drug/pre-drug) in each trace and then summarized and compared the ratio values (Kohno et al., 2005). The results showed that EM2 caused a lower decrease in the frequency of sEPSCs in diabetic rats than in control rats (*p* < 0.05, unpaired Student’s *t*-test, Figure 7G). At the end of the recording, bath application of 10 μM 6-cyano-7-nitroquinoxaline-2,3-dione (CNQX, AMPA receptor antagonist) blocked the frequency and amplitude of sEPSCs of the neurons (*n* = 3/group, data not shown), which further confirmed that the sEPSCs were glutamate-mediated postsynaptic currents.

## Discussion

### Decreased EM2 and MOR in the Spinal Dorsal Horn are Associated with Progressive PDN in Diabetic Rats

The distributions of EM2-ir structures, including neuronal cell bodies, dendritic processes, axonal fibers and terminals, in the nervous system have been studied extensively (Hosohata et al., 1998; Martin-Schild et al., 1999). In contrast to EM1-ir structures, which are predominantly located in the brain, EM2-ir structures are mainly observed in the spinal cord (Hui et al., 2010). On the other hand, when intrathecally injected, EM2 plays more efficient analgesic roles than EM1 does (Tseng et al., 2000). Our immunochemical findings showed that EM2-ir terminals were densely located in the superficial laminae (laminae I and II) of the spinal dorsal horn. Moreover, the expression of EM2 decreased significantly in diabetic rats, which was consistent with the development of mechanical allodynia from day 14–28. We also used ELISA to detect EM2 concentrations in the spinal cord, and the results confirmed that the reduction of EM2 in the spinal cord was in line with the progressive PDN in diabetic rats.

The next stage was to consider the relationship between PDN and decreased EM2 in the spinal dorsal horn in diabetes. We found that EM2 levels were correlated with progressive mechanical allodynia from day 14–28; the EM2 concentration decreased significantly in the brain, lumbar spinal cord and CSF of diabetic rats. However, in contrast to EM2 changes in the brain, the changes in EM2 concentrations in the spinal cord and CSF occurred as early as day 3 in the diabetic model, indicating that diabetes affects EM2 at the initial stage of PDN in the spinal cord. Previous reports indicate that in diabetic patients and animals, poor blood glucose control might result in the reduced inhibition of EM2 in gastrointestinal disorders (Wang et al., 2014). EM2-immunopositive (IP) terminals in the spinal dorsal horn principally originate from the central axons of the attached DRGs (Hui et al., 2010), which belong to the peripheral nervous system (PNS) and are thus more sensitive to high glucose in the periphery. This peripheral localization of the DRGs might be the most important reason among all of the possible reasons for the early decreased EM2 levels in the spinal cord. We noted that the tendency for reduced EM2 concentrations could be found in the plasma in diabetic rats; however, statistical analyses suggest that there was no significant difference among groups. We chose the early stage (14–28 days) of diabetes in the present study to investigate the mechanism of PDN; therefore, the unchanged EM2 in the plasma may be associated with a time delay. These results indicate that the reduction in EM2 levels in the spinal dorsal horn occurs at the early stage of diabetes, which might be correlated with the progressive PDN.

### Presynaptic MOR may be Involved in the Decreased Inhibition of EM2 in the Spinal Dorsal Horn Related to PDN

MOR is the principal endogenous receptor in the spinal cord that responds to EM2 and is involved in the antinociception induced by EM2. Our results indicate that, similar to EM2 distribution, the expression of MOR was concentrated in the superficial layer of the spinal dorsal horn (Zadina et al., 1997; Wang et al., 2003; Greenwell et al., 2007). In diabetic rats, our immunohistochemistry analyses provided evidence that the expression of MOR was reduced significantly, along with the decrease in EM2 expression in the spinal dorsal horn, indicating that MOR may be involved in antinociception produced by EM2 in PDN.

In the spinal cord, EM2 binds and activates MOR at both pre- and postsynaptic sites to drive Gαi/o protein, leading to the blockade of Ca^2+^ channels and/or the activation of K^+^ channels, the suppression of transmitter release and membrane hyperpolarization (Fichna et al., 2007; Sesena et al., 2014; Chen et al., 2015b). Particularly at presynaptic sites, EM2 can decrease the excitability of neurons and inhibit the release of glutamate (Glu), substance P (SP) and calcitonin gene-related peptide (CGRP) through the activation of presynaptic MOR on primary afferent fibers in the spinal dorsal horn (Fichna et al., 2007). Based on patch-clamp recording, our results showed that the frequency of glutamatergic sEPSCs was significantly higher in diabetic rats than in control rats, suggesting that the glutamate release from the primary afferents that acts on neurons in the spinal dorsal horn may increase on day 14, which may contribute to the hyperexcitability of the dorsal horn neurons in PDN. We found that EM2 inhibited sEPSCs less in diabetic than in control rats; hence, in response to EM2, MOR activity at the primary afferent terminals may be down-regulated in PDN. In contrast to frequency, there was no significant difference in the amplitude of sEPSCs between diabetic rats and controls. Therefore, compared to postsynaptic receptors, presynaptic MORs may play a more important role in the regulation of nociception and antinociception exerted by EM2 on neurons in the spinal dorsal horn.

In the spinal dorsal horn, MORs localized at presynaptic sites are mainly derived from the central axons of the DRG (Zhou et al., 2014; Honsek et al., 2015), and therefore, we investigated the changes in MOR expression by Western blotting in the spinal cord and the DRG. The results indicate that from day 14, the protein levels of MOR were reduced significantly in diabetic rats in both the spinal cord and the DRG. Thus, rather than postsynaptic MORs, presynaptic MORs may exert a major effect on the inhibition of EM2 in the spinal dorsal horn related to PDN.

### EM2 Treatment Partially Alleviates PDN at the Early Stage of Diabetes

Previous reports suggest that EM2 induces MOR endocytosis and may act as a feedback mechanism to activate MOR mRNA transcription, whereas morphine treatment decreases MOR membrane density (McConalogue et al., 1999; Yu et al., 2003). Therefore, we delivered EM2 intrathecally as a treatment for 14 days starting with day 14 and observed the effects of EM2 on behavioral tests and MOR activity in diabetic rats. Our results demonstrate that the intrathecal injection of EM2 could significantly alleviate the mechanical allodynia in diabetic rats, suggesting that EM2 treatment may be an effective way to alleviate PDN at the early stage of diabetes.

Moreover, after EM2 treatment, diminished MOR expression was partially recovered in the spinal cord. Previous reports suggest that endogenous opioid peptides stimulate a selective phosphorylation of the carboxyterminal residue 375 (Ser^375^; Schulz et al., 2004; Doll et al., 2011; Grecksch et al., 2011). Application of EM2 effectively attenuated the decreased pMOR (Ser^375^) in diabetic rats on day 14, suggesting that the attenuated MOR activity may be associated with EM2 application.

## Conclusion

In summary, our findings showed a reduction in EM2 and MOR expression in the spinal dorsal horn in STZ-induced diabetic rats with progressive mechanical allodynia. The diminished EM2 and reduced MOR activity at presynaptic sites in the spinal dorsal horn may be involved in the early stage of the development of PDN.

## Author Contributions

Z-ZK, HL and Y-QL conceived and designed the experiments. Z-ZK, F-PW, YB, C-YL, J-CH and G-TZ performed the experiments and acquired data. TZ, TC and Y-YW analyzed the data. Z-ZK, HL and Y-QL wrote the article.

## Conflict of Interest Statement

The authors declare that the research was conducted in the absence of any commercial or financial relationships that could be construed as a potential conflict of interest.
